# Sequential adjustment of cytotoxic T lymphocyte densities improves efficacy in controlling tumor growth

**DOI:** 10.1038/s41598-019-48711-2

**Published:** 2019-08-23

**Authors:** Roxana Khazen, Sabina Müller, Fanny Lafouresse, Salvatore Valitutti, Sylvain Cussat-Blanc

**Affiliations:** 1 Centre de Recherches en Cancérologie de Toulouse (CRCT), UMR 1037 INSERM/ Université Toulouse III Paul Sabatier, «Equipe labellisée Ligue Nationale contre le cancer 2018», INSERM, Toulouse, France; 2 Present Address: INSERM U1223, Dynamics of Immune Responses Unit, Institut Pasteur, 75015 Paris, France; 3 Department of Pathology, Institut Universitaire du Cancer-Oncopole de Toulouse, 31059 Toulouse, France; 4 Institute of Advanced Technologies in Living Sciences, CNRS – USR3505, Toulouse, France; 5 University of Toulouse, Institute of Research in Informatics of Toulouse, CNRS – UMR5505, Toulouse, France

**Keywords:** Immunology, Immune evasion

## Abstract

Understanding the human cytotoxic T lymphocyte (CTL) biology is crucial to develop novel strategies aiming at maximizing their lytic capacity against cancer cells. Here we introduce an agent-based model, calibrated on population-scale experimental data that allows quantifying human CTL per capita killing. Our model highlights higher individual CTL killing capacity at lower CTL densities and fits experimental data of human melanoma cell killing. The model allows extending the analysis over prolonged time frames, difficult to investigate experimentally, and reveals that initial high CTL densities hamper efficacy to control melanoma growth. Computational analysis forecasts that sequential addition of fresh CTL cohorts improves tumor growth control. *In vivo* experimental data, obtained in a mouse melanoma model, confirm this prediction. Taken together, our results unveil the impact that sequential adjustment of cellular densities has on enhancing CTL efficacy over long-term confrontation with tumor cells. In perspective, they can be instrumental to refine CTL-based therapeutic strategies aiming at controlling tumor growth.

## Introduction

Various strategies are currently employed to potentiate human CTL-mediated immune responses in cancer patients with the goal of impairing tumor progressions. Although, *in vitro* studies put forth the notion that human CTL are very sensitive to antigenic stimulation and elicit rapid cytotoxicity responses^1–4^, it is known that tumor cells exhibit various mechanisms of resistance to CTL attack^5,6^ and that the tumor microenvironment is plagued by multiple mechanisms of immune cell inhibition^7,8^. It is therefore critical to develop an optimized metrics of human CTL efficacy against cancer cells. A better metrics of CTL responses might be instrumental to adapt therapeutic strategies based on CTL adoptive transfer and limit tumor escape from immune surveillance^9^.

In this context, a key unresolved question concerns the definition of human CTL per capita killing rates. Understanding the inner dynamics of human CTL/tumor cell interaction is indeed crucial to define how efficient individual CTL are when facing cancer cells for a prolonged time. Unfortunately, gaining *in vitro* data on human CTL per capita killing requires visualization of a large number of individual CTL/target cell interactions using time-lapse microscopy optimized for single cell inspection^10–12^. This approach is technically challenging and complex to extrapolate to clinical settings.

To overcome such a technical bottleneck, we designed an agent-based model, calibrated on experimental measurements, which accurately reproduces human CTL/tumor cell interactions taking place within the culture dish. This *in silico* approach allows to perform data analysis and modeling of *in vitro* results obtained at the population level and to extrapolate them to the single cell level. It also allows to dissect individual cell behaviors that could not be inferred by standard *in vitro* assays. Furthermore, various complex scenarios of CTL/target cell interaction (e.g. cytotoxicity assays lasting several days, investigation of a large spectrum of effector/target (E/T) ratios, etc.) can be explored *in silico*.

Computational studies addressing the behavior of CTL and target cells during their competitive interaction *in vitro* and *in vivo* have been previously released. Indeed, complex multi-step immune cell/tumor cell interactions within the highly heterogeneous tumor microenvironment can be mathematically modelled as predator/prey dynamical systems in which several global parameters of the immune response can influence the success of one of the two populations over the other^13–17^. In the present study aiming at defining CTL per capita killing, we focus our investigation on the events occurring at the contact site between CTL and target cells during the very rapid process of lethal hit delivery^18–20^.

Our *in silico* model provides several lines of evidence that extend experimental results. First, it reveals CTL super-killing activity within the whole population, thus supporting previously reported experimental data obtained at the single cell level^10^. Second, it points out that CTL per capita killing is higher at lower E/T ratios. Third, it highlights a global weakness of CTL to control tumor growth over prolonged time even at high E/T ratios. Finally, an important prediction of the model is that sequential addition of fresh CTL cohorts improves CTL efficacy in controlling tumor growth. We implemented this protocol experimentally and obtained *in vivo* results that confirm model prediction.

## Results

### High cell densities limit the per capita CTL efficacy during prolonged interaction with target cells

In order to investigate whether and how individual CTL in a whole population might influence each other in their capacity to kill target cells, we designed an experimental protocol allowing to study killing efficacy during overnight interaction at very low E/T ratios. Human CTL were conjugated with target cells (either conventional target cells such as EBV-B cells or melanoma cells) pulsed with the antigenic peptide or left unpulsed. Cytotoxicity was measured by FACS analysis.

**Figure 1:**
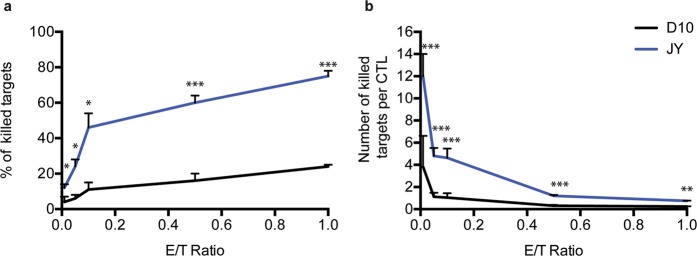
Density of CTL negatively correlates with their individual capacity to kill target cells. Population scale (**a**) and per capita (**b**) killing efficacy of human CTL during overnight interaction with melanoma cells or conventional target cells. Target cells were pulsed with the antigenic peptide and co-cultured for 18 hours with antigen-specific CTL at the indicated E/T ratios. Cytotoxicity was evaluated by flow cytometry using 7-AAD uptake by target cells. Cytotoxicity is expressed as fold increase over corresponding basal death. Data are from at least three independent experiments and are represented as mean ± SD. Unpaired student’s t-test using the GraphPad Prism software (version6; GraphPad) was used to determine the statistical significance of differences between groups. ***P < 0.001, **P < 0.0, *P < 0.05.

In line with our previously reported data^5,10,21^, we observed that, while killing of conventional target cells could be detected even at very low E/T ratios (<0.5) when a sustained interaction time was allowed, melanoma cells were relatively resistant to CTL attack for a prolonged time (Fig. 1a).

The analysis also provided the intriguing result that the per capita CTL killing efficacy was much higher at low E/T ratios when compared to high E/T ratios. Figure 1b shows the average per capita killing *k*_*ctl*_ calculated with the following formula:

$${k}_{ctl}=\frac{{n}_{kt}}{{n}_{ctl}}$$

where ${n}_{kt}$ is the number of target cells killed and *n*_*ctl*_ is the number of CTL. Results show that at 0.01 E/T ratio each CTL could kill on average 12 conventional target cells and 3.3 melanoma cells while the per capita killing rates sharply decreased with increasing E/T ratios.

Taken together, the above results indicate that the density of CTL negatively correlates with their individual capacity to kill target cells.

### An agent-based model reproduces cytotoxic activity with unprecedented resolution

**Figure 2:**
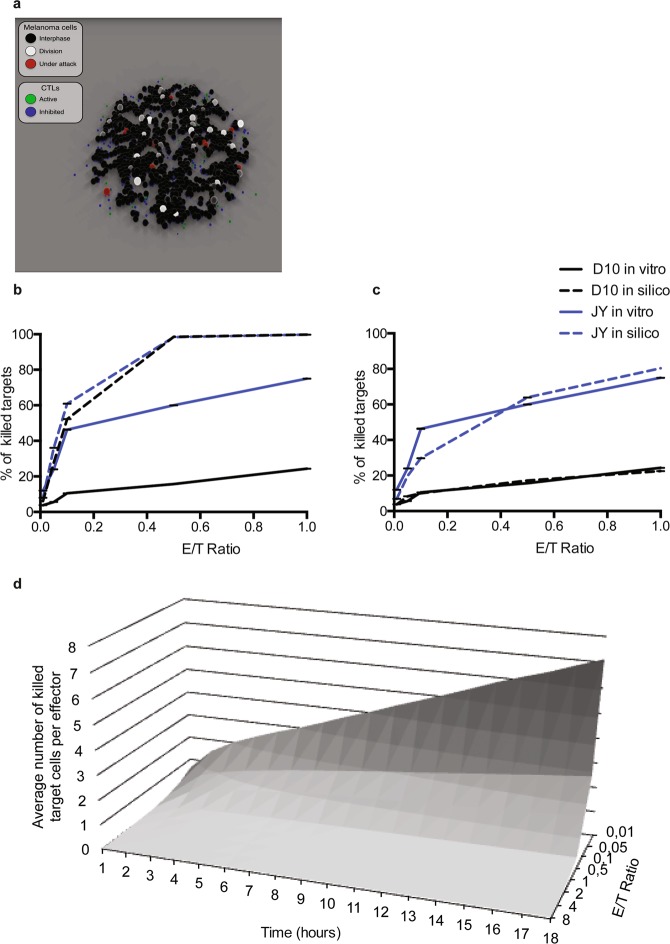
Simulation of killing assays and detailed temporal analysis of per capita killing efficacy using an agent-based model. Panel (**a**) shows a snapshot taken from a simulated interaction at 1:1 E/T ratio (see Supplementary Movie 1). (**b**,**c**) Comparison of *in vitro* and *in silico* data of killing efficacy either without (**b**) or with (**c**) probabilistic inactivation of CTL during *in silico* simulations. (**d**) Temporal statistics of per capita CTL killing activity accessible using the computational model. Results shown in (**b**,**c**) correspond to mean +/− SD of 20 independent runs. Results in (**d**) correspond to mean of 20 independent runs.

We next built an agent-based model that could reproduce the behavior of both CTL and target cells within an *in silico* set up. As shown in Movies 1 and 2 and Fig. 2a, the computational model qualitatively re-created the *in vitro* experimental conditions at different E/T ratios within a virtual culture well. Once calibrated, the model was then used to evaluate various CTL/target cell interaction scenarios. (Model description is provided in Methods Section and Supplementary Fig. 1. Parameters used in this study are given in Supplementary Table 1).

Interestingly, Fig. 2b,c show that the model was able to quantitatively fit experimental data only if a probabilistic inactivation of CTL was implemented during the cytotoxicity process. The model also allowed a detailed temporal monitoring of the culture well by providing “real time” statistics of per capita CTL killing activity. It showed increasing killing efficacy at low CTL densities (Fig. 2d).

**Figure 3:**
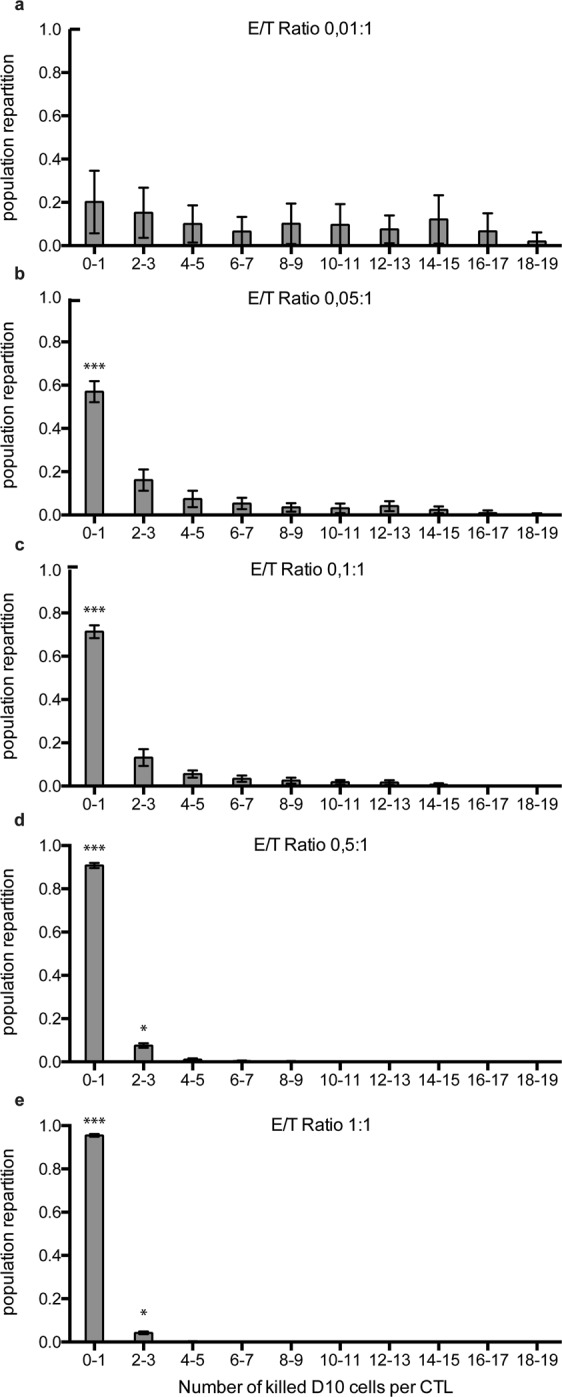
Computational modeling highlights deficiency of individual CTL killing capacity at high CTL densities. The panels show the population repartition of CTL killing efficacy at different E/T ratios. The fraction of CTL within the whole population killing a high number of melanoma cells increases with the decrease of the E/T ratio. The E/T ratios simulated in the model are indicated in panels. Results correspond to mean +/− SD of 20 independent runs.

Further analysis showed that at very low CTL/melanoma cell ratios (Fig. 3a–c) a large fraction of the CTL population killed more than 5 target cells with few CTL killing up to 17–18 target cells. At higher E/T ratios (Fig. 3d,e) this fraction progressively decreased and disappeared. Similar results were observed with conventional target cells although, under these conditions, the global numbers of killed targets were higher (Supplementary Fig. 2).

The above observations are in line with our previous experimental results, obtained at single cell level, showing that a fraction of CTL, within a clonal population, behaves as highly efficient killer cells by killing more than 10 target cells^10^. The present computational results extend our previous data by showing that super-killer behavior is more pronounced at very low E/T ratios.

Taken together the above results show that our model accurately reproduces human CTL killing assays *in silico*. In line with experimental data, the model supports the existence of an intrinsic mechanism controlling CTL-mediated cytotoxicity. Furthermore, the above results allow retrieving individual cell behaviors starting from experimental data that were obtained in population.

### CTL are intrinsically inefficient in controlling tumor cell growth over prolonged time

**Figure 4:**
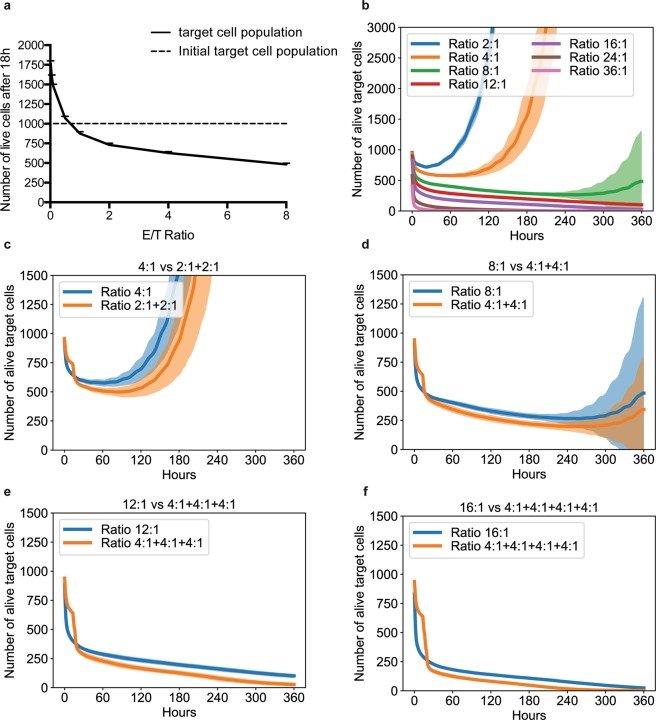
Prolonged time simulations show an intrinsic weakness of CTL in controlling tumor growth that can be overcome by sequential addition of fresh CTL cohorts. Panel (**a**) shows the number of target cells alive after 18 hour simulated co-culture at different E/T ratios. Panel (**b**) shows the limited control of tumor growth during 15 days of simulated co-culture. Panels (**c–f**) show the benefit of multiple additions of fresh CTL cohorts to the simulated culture. Results correspond to mean +/− SD of 20 independent runs. Statistical comparison of data shown in panels (**c–f**) was performed using Wilcoxon test and is presented in Supplementary Table 2.

The experimental and computational results showing that CTL density inversely correlates with individual cell killing efficacy prompted us to look at the “optimal E/T ratio” sufficient to control tumor target cell growth without using an excess of CTL. As shown in Fig. 4a, at an E/T cell ratio close to 1:1, an efficient control of the tumor growth was observed over a period of 18 hours. More precisely, the optimal ratio for 18 hour simulations was 0.615 E/T ratio (with 999.65 target cells remaining after 18 hour interaction with CTL, averaged on 20 runs, std = 27.2653144). This value has been obtained by parameter sampling with 0.005 steps between 0.5 and 1 E/T ratio. To verify whether this optimal E/T ratio would be effective also at longer times of interaction, we performed computer–assisted simulation of CTL/target cell interactions lasting up to 360 hours (15 days), a time incompatible with classical experimental killing assays. This analysis showed that tumor cells tended to outnumber CTL during prolonged time (Fig. 4b). Indeed, statistical analysis of multiple simulations showed that, even at E/T ratios as high as 16:1, a small fraction of melanoma target cells was still alive. A very high E/T ratio (>24:1) was necessary to efficiently control tumor growth over 360 hours.

Together, the above results indicate that while the “optimal E/T ratio” can provide a score for the killing efficacy of a given CTL population within a defined time window, it is not an absolute value indicating the number of CTL required to durably control the growth of a given target cell population. Indeed, the capacity of CTL to control resistant tumor target cells over a prolonged time is limited even at high E/T ratios.

### Sequential addition of fresh CTL cohorts improves CTL efficacy and allows long-term tumor growth control

We employed our computational model to explore various CTL/tumor cell interaction scenarios with the aim of improving protocols allowing tumor cell growth control over a prolonged time. Since our experimental and computational observations revealed that high cell densities limit the per capita CTL efficacy, we investigated the possibility that deferring the interaction between CTL and tumor cells might enhance tumor control. To this end, we simulated the sequential addition of fresh cohorts of CTL at different time intervals. As shown in Fig. 4c–f, at equal numbers of CTL added, we observed a better control of tumor growth if CTL were added sequentially instead of being all present at the beginning of the culture. Supplementary Table 2 statistically confirms the previous analysis by the mean of a Wilcoxon test comparing the efficacy in tumor cell control exerted by equivalent numbers of CTL either all present at time 0 or sequentially added: p-values of each comparison are lower  than 10^−2^.

All in all, the above-presented computational data revealed a clear advantage of protocols involving sequential CTL addition during prolonged time over protocols based on initial large cohorts of CTL.

We intended to experimentally verify this forecast, however to reproduce such scenarios *in vitro* was technically challenging because of the impossibility to perform *in vitro* killing assays lasting up to 10–15 days. To overcome this limitation and experimentally evaluate the model forecast, we investigated the impact that sequential addition of antigen-specific CTL might have on the control of sub-cutaneous B16 mouse melanoma nodules. This approach was chosen to analyze tumor control over a time window comparable to that of the computational model and to verify model prediction in an *in vivo* set up.

**Figure 5:**
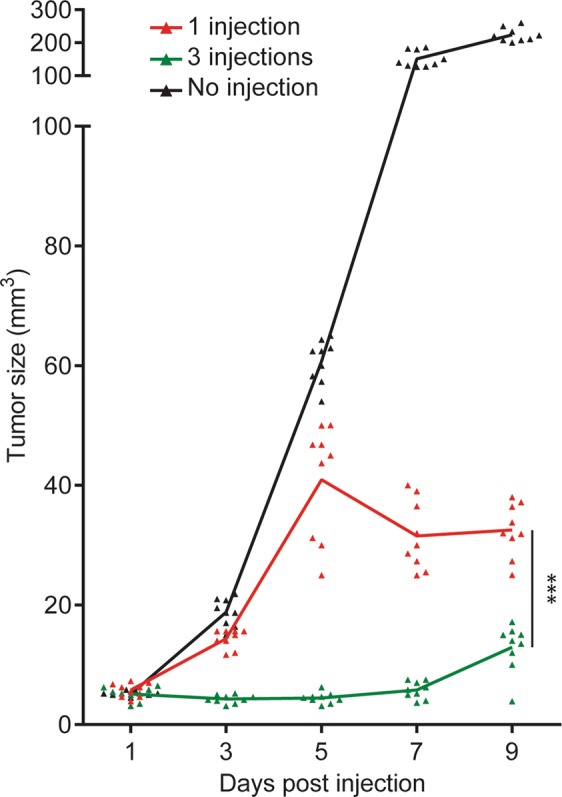
Sequential addition of fresh CTL cohorts results in better control of tumor growth *in vivo*. C57BL/6 mice were injected subcutaneously with 2 × 10^6^ OVA^+^ B16 tumor cells. On day 5, mice were adoptively transferred with 6 × 10^6^ activated OT-I CD8^+^ T cells either in 1 injection or in 3 injections of 2 × 10^6^ each with a time interval of 4 hours. A third group served as control with no CTL injection. Tumor growth was monitored over time. Data are from three independent experiments performed on 3 mice per group and are represented as mean +/− SD. Data was analyzed by two-way repeated measures ANOVA using the GraphPad Prism software. ***P < 0.0001.

As shown in Fig. 5, sequential CTL addition resulted in a significant enhancement of tumor growth control compared to initial injection of an equivalent number of CTL.

Taken together, our results reveal that the pace at which antigen-specific CTL cohorts join the tumor microenvironment has a major impact on the efficacy of tumor elimination.

## Discussion

In the present study, we release a new computational tool calibrated on experimental data that describes the inner dynamics of competitive CTL/tumor cell interaction by zooming into a virtual culture dish with an unprecedented resolution. This tool allows to rapidly explore a broad range of cell/cell interaction scenarios and provides a stepping-stone to inspire strategies enhancing CTL efficacy over prolonged time frames. We report that fine-tuning of killer cell density during prolonged interaction with target cells is of central importance in enhancing tumor cell control by CTL.

An important aspect of our interdisciplinary study is that it developed as teamwork. Biologists and computer scientists designed initial experiments while experimental results alimented the computational model. In turn, computational model predictions inspired further experiments.

To investigate the efficacy of CTL in killing conventional or tumoral target cells, we used, as cellular models, EBV-B cells and melanoma cells that were either pulsed with strong antigenic ligands (peptides of the human cytomegalovirus protein pp65) or left unpulsed before conjugation with cognate CTL. This strategy was chosen to avoid the possibility that obtained results could be biased by sub-optimal CTL activation when facing tumor target cells. Virus specific CTL are indeed fully activated to lethal hit delivery when interacting with peptide-pulsed melanoma cells^5,21^.

More than 20 years ago, others and ourselves put forth the notion of the exquisite sensitivity of T cell antigen recognition. It is now well established that T lymphocytes can be activated by an extremely small number of specific peptide/MHC complexes displayed on the antigen presenting cell surface^22–24^. In human CTL, we showed that lethal hit delivery has an extraordinarily low threshold since it can be saturated at concentrations of antigenic peptide used for pulsing much lower ([pM/nM] concentrations^2^) than those required to activate CTL to IFN-γ production ([μM] concentrations). More recently, we put forth the notion of lytic and stimulatory synapses formed by CTL when interacting with peptide pulsed target cells. We showed that CTL can form lytic synapses and exhibit saturating cytotoxicity responses when interacting with target cells pulsed with very low antigen concentrations. Conversely, CTL require a much stronger antigenic stimulation to form stimulatory synapses and to be activated to IFN-γ production^3^.

M.M. Davis and coworkers substantially contributed to this field by employing methods allowing to directly visualize peptide/MHC complexes present at the immunological synapses formed by both helper T cells and CTL. Their seminal studies showed that both helper and cytotoxic T cells activate their biological function when detecting as few as 3 to 12 specific peptide/MHC complexes at the immunological synapse^4,25,26^.

The notion that human CTL exhibit an extremely low threshold for activation to lethal hit delivery and are fully activated to cytotoxicity when interacting with peptide-pulsed target cells allowed us to implement in our computational model a scenario in which each initial encounter between a CTL (still in its activated state) and a target cell would result in productive triggering of cytotoxic response against that specific target.

The agent-based model designed in this study appears to be a valuable tool to improve the comprehension of the complex interactions taking place during CTL-mediated cytotoxicity since it allows to extract data on individual cell behaviors from standard experimental protocols performed at whole population scale.

Although equation-based models can valuably represent cellular interactions and have been successfully employed to describe several aspects of the immune response including CTL-mediated target cell cytotoxicity^27–29^, we preferred an agent-based model since such an approach is flexible and easy to adapt to different experimental scenarios^30–32^. Another important feature of our approach is that it is accessible to biologists who can therefore actively participate to the model design. Such a synergy is crucial to make sure that generated models are compliant with the observed reality. Also, when compared to standard cellular automata^33^, the use of continuous virtual environment allows finer simulation of cell mobility and, consequently of cell-cell dynamic interactions, which are the corner stone of cytotoxicity. Although the model could have allowed us to study spatial dynamics, we mainly focused on CTL density rather than on the characteristics of cell motility and interactions. This choice was justified by the necessity of creating a computational scenario as adherent to our experimental protocol as possible. Finally, we paid a particular attention to calibrate the model all-along its development with detailed experimental data. This procedure led to the emergence of realistic cell behaviors at the whole population scale and, in turn, to obtain accurate forecasts of the outcome of cell-cell interactions.

An unresolved question in CTL biology concerns the definition of the per capita killing efficacy at the single cell level under different CTL/target cell interaction scenarios. Although recent studies described protocols to measure individual CTL killing rates both *in vitro* and *in vivo*^10,12,34^, measurements of killing capacity at the single cell level are technically challenging and difficult to adapt to different experimental conditions. Having validated the capacity of the model to accurately reproduce CTL/target cell behaviors and to mimic different interaction scenarios, we used our computational analysis to estimate the per capita killing efficacy of the individual cells within the virtual culture plate.

Another advantage of our model is that it can be extended to the third dimension to mimic different scenarios of 3-D CTL/target cell interaction, thus providing a description of cellular interactions closer to those occurring *in vivo* without the need of performing complex 3-D imaging experiments.

A first observation that stemmed from our *in silico* reproduction of CTL/target interactions was that the model could not fit experimental data without implementing an inhibitory mechanism of the CTL killing activity, which is in agreement with previous computational analyses and is therefore somehow expected^13,14,35,36^. However, it is interesting to note that in our system, the observation that CTL density has a negative impact on their cytotoxic function, comes mainly from our experimental data (Fig. 1), while the computational analysis permitted us to extend experimental data.

The observation that in the absence of a probabilistic inactivation of CTL the difference in resistance to cytotoxicity between conventional target cells and melanoma cells (well documented *in vitro*) is rapidly abolished *in silico* might appear intriguing (Fig. 2b). However, it should be noted that, with the increase of E/T ratios, this difference tends to be progressively reduced also in experimental conditions^21^. At E/T ratios as high as 10:1 to 20:1, the number of target cells that can be killed is expected to converge towards values close to 100% for both conventional target cells and melanoma cells (see blue line in Fig. 4f depicting a 16:1 E/T ratio). In the absence of probabilistic inactivation of CTL this process is dramatically enhanced. For this reason the percentage of killed cells rapidly reaches 100% in both cell types also at low E/T ratios.

The molecular nature of such inhibitory mechanism(s) is presently elusive. It is tempting to speculate that inhibitory metabolites might be released by dying target cells or by activated CTL to locally extinguish the killing activity. The observed limitation of cytotoxic function at high cell density appears to be independent from the nature of the employed target cells and to be an intrinsic characteristic of CTL. Indeed, we observed a similar trend in CTL per capita killing both with melanoma cells and conventional target cells. Even though this mechanism has to be further experimentally explored, its description might be of major importance in understanding regulation of CTL-mediated immune responses.

The model we propose can potentially be upgraded into new models based on clinical data that might be instrumental to evaluate experimental protocols aiming at improving the killing efficacy of CTL in patients. An example of the potential of our combined experimental and computational approaches is our observation that progressive adjustment of CTL densities might enhance cytotoxic activity. We show *in silico* and confirm *in vivo* that sequential re-addition of fresh CTL cohorts is more efficient to control tumor growth when compared to a unique initial CTL cohort. It is interesting to speculate that these results might have relevance for CAR-T cell therapeutic strategies. Although these therapies are very promising and are providing objective clinical amelioration in a fraction of patients affected by hematological malignancies, they need optimization. In particular, the dosing strategies might be adjusted to provide a longer duration of CAR-T cell therapeutic effect while limiting the adverse effects of the treatment^37^. More generally, our results obtained in a binary cellular model, might inspire new research aiming at defining per capita killing in clinical settings by exploring more complex scenarios of CTL-mediated tumor immune-surveillance.

Since, the model is effective to forecast long-term outcomes of CTL/tumor cell interactions, it is tempting to speculate that the model might be instrumental to evaluate more complex scenarios in which additional cell types, known to modulate immune surveillance against tumors (such as tumor associated macrophages, regulatory T cells, etc.), might be introduced in order to evaluate their quantitative contribution to tumor escape. Further experimental and computational research will be required to address this important point. In order to envisage modelling complex scenarios in immunotherapy, it will be necessary to experimentally measure the impact that “additional players” of the immune response might have on our binary cellular model and then progressively enrich the complexity of the model with new data generated from specifically designed experiments.

So far, several computational studies addressed various aspects of the complex dynamics of immune surveillance against cancer. For instance, Alfonso *et al*. used agent-based modeling to simulate the inflammatory process in breast tissues which involves many types of cells (myoepithelial, luminal, effectors and regulators) acting in a 2-D discrete environment^36^. Authors used both data from literature and data extracted from whole-slide imaging immunohistochemistry to calibrate the model and were able to improve the prognostic of the patients based on analysis performed using the model. In Hatzikirou *et al*.^13^, the authors propose an equation-based framework at the cell population scale to analyze the impact of immune recruitment via vascularization in tumors and propose a new therapeutic approach for personalized tumor profiling. To this end, the model’s parameters are explored in order to study the behavior of the simulated tumor-effector system in various conditions leading to the conclusion that an optimal effector concentration range exists in order to better control tumor growth. In line with our paper, D’Onofrio *et al*. proposed a general mathematical framework that models the tumor-immune system competition and proposes new therapeutic strategies based on periodic re-addition or infusion therapies^14^. The novelty and the peculiarity of our study, when compared to previous ones, consists in our strategy to focus the investigation essentially on the events occurring at a well-defined step of CTL fight against tumor target cells: lethal hit delivery^18–20^. We accumulated over the years a profound knowledge about human CTL activation and cytotoxic responses^10,18,19,21^. This allowed us to feed the model with parameters that were all measured experimentally. The combined results from experimental and computational strategies, unexpectedly revealed that the density of CTL can influence their per capita killing independently of signals derived from additional components of the immune system.

Although the approach we chose in our study might appear simplistic, it has the advantage of allowing constant cross-fertilization among experimental and computational approaches. Moreover and importantly, our approach is justified by a considerable amount of recently published data showing that, in clinical settings, the number of CD8^+^ T cells present in the tumor microenvironment and their activation status, are major parameters in predicting disease progression and response to immunotherapy in cancer patients^38,39^. There is therefore strong need to combine experimental and computational efforts to better understand the process of CTL-mediated cytotoxicity.

In conclusion, our study releases a computer model that reproduces *in silico* cytotoxicity assays and contributes to a better understanding of the fine balance between human CTL efficacy and tumor target cell resistance measured in cytotoxicity assays. As above-discussed, our findings might be instrumental for translational studies aiming at establishing/improving personalized therapeutic protocols based on the potentiation of CTL efficacy against cancer.

## Material and Methods

### Cells and mice

Human T cell clones and target cells were generated and maintained as described in^5^ and as detailed below. Human CD8^+^ Tcell lines were purified from healthy donor blood samples using the RosetteSep Human CD8^+^ T Cell Enrichment Cocktail (StemCell Technologies). For cloning, HLA-A2-restricted CD8^+^ T cells specific for the NLVPMVATV peptide or the VLAELVKQI peptide of the cytomegalovirus protein pp65 were single cell sorted into 96-U-bottom plates using a BD FACSAria II cell sorter using tetramer staining. Cells were cultured in RPMI 1640 medium supplemented with 8% human AB serum (PAA), minimum essential amino acids, HEPES and sodium pyruvate (Invitrogen), 100 IU/ml human rIL-2 and 50 ng/ml human rIL-15. CD8^+^ Tcell clones were stimulated in complete RPMI/HS medium containing 1 μg/ml PHA with 1 × 10^6^ per ml 35 Gy irradiated allogeneic peripheral blood mononuclear cells (isolated on Ficoll Paque Gradient from buffy coats of healthy donors) and 1 × 10^5^ per ml 70 Gy irradiated EBV-transformed B cells. Re-stimulation of clones was performed every 2–3 weeks. Blood samples were collected and processed following standard ethical procedures (Helsinki protocol), after obtaining written informed consent from each donor and approval by the French Ministry of the Research (AC-2014-2384).

The following HLA-A2^+^ cell lines were used as target cells: EBV-transformed B cells (JY) and D10 cells (isolated from metastatic melanoma patients, kindly provided by Dr G. Spagnoli, Basel, Switzerland^5^).

To determine target cell division rate, live target cell number was measured by manually counting cells in the culture wells using a Neubauer chamber and trypan blue dye exclusion, every 24 hours. JY and D10 cultures were respectively started with 0.25 × 10^6^ and 0.9 × 10^6^ cells per well. In parallel experiments, cells were fixed and permeabilized in 70% ethanol for 30 min on ice and stained with DAPI at 1 μg/ml (Sigma D9542), followed by FACS analysis. The duration of cell cycle phases was estimated using a mathematical workflow described in Bernard *et al*.^40^.

B16 mouse melanoma cell line was a kind gift from Dr. P. Bousso (Institute Pasteur, Paris).

6-8-week-old C57BL/6 mice were purchased from Charles River. OT-I TCR, Ubi-GFP, Ubi-GFP RAG1^−/−^ OT-I TCR mice were bred in the animal facility of the Institut Pasteur under specific-pathogen free conditions. All animal studies were approved by the Pasteur Institute Safety Committee in accordance with French and European guidelines (CETEA 2013-0077). Mice were examined every day and sacrificed in case of prostration, tousled-hair or weakness.

### Cytotoxic assay

Cytotoxicity assay was performed as described in^5^ and as detailed below. Target cells were left unpulsed or pulsed with 10 μM antigenic peptide during 2 h at 37 °C/5% CO_2_, washed three times and subsequently transferred to a 96-well U-bottom plate at 25 × 10^3^ cells per 100 μl RPMI, 5% FCS/HEPES. CTL were previously stained with 0.1 μM CMFDA for 15 min at 37 °C/5% CO_2_, washed and added to the target cells at indicated CTL/target cell ratio in 100 μl RPMI, 5% FCS/HEPES. Cells were pelleted for 1 min, 455 *g* and incubated at 37 °C/5% CO_2_ for the indicated time points. Before FACS analysis, 0.25 μg 7-aminoactinomycin D (7-AAD; BD Biosciences) was added to each sample to measure the percentage of dead targets.

### *In vivo* tumor growth

OVA expressing B16 Tumor cells were harvested at exponential phase, and 2 × 10^6^ tumor cells were re-suspended in 50 μl of PBS and injected subcutaneously in mouse leg.

Mouse CD8^+^ T cells were purified from the lymph nodes of Ubiquitin C-GFP × OT-I TCR Tg mice using the depleting CD8 isolation kit (Milteny) and were activated in plates coated with 2.5 µg anti-CD3 mAb (clone 17.A2) in the presence of 2.5 µg/mL soluble anti-CD28 mAb (clone 37.51) for 48 hours. T cells were then cultured for 3–4 additional days in the presence of 25 IU/mL rIL-2 (Roche).

Recipient mice were adoptively transferred with 6 × 10^6^ purified *in vitro* activated OT-I CD8^+^ T cells on day 5 post tumor injection either in a single injection of 6 × 10^6^ cells or three injections of 2 × 10^6^ cells every 4 hours. Tumor volumes were recorded every 2–3 days. These methods were adapted from^41^.

### Model description

We used agent-based modeling to simulate interactions between target cells and CTL ongoing into a culture dish. In the model, individual cells are represented by independent agents. They can be of two types: target cells or CTL. Cell behaviors have been designed thanks to extensive interaction between computer scientists and immunologists with the aim to capture the biological knowledge of the complex system to simulate. State-transition diagrams, shown in Supplementary Fig. 1, illustrate the resulting cell behaviors. Time is discretized with a ${\rm{\Delta }}t$ time steps, chosen small to improve mechanical stability of the simulation. Cells are acting in a virtual continuous physical world in which cell-cell membrane adhesions are simulated with mass-spring-damper system. Collisions and cell-cell adhesion are simulated. The virtual culture well is initialized with cells of both types (numbers depend on E/T ratio), all aggregated in the center of the dish to simulate centrifugation. The aim is indeed to carefully reproduce the centrifugation step employed in the *in vitro* experimental protocols. All parameters used in simulation are provided in Supplementary Table 1.

#### Target cells

When created, target cells start cycling. At the beginning, durations of the target cell cycle phase are randomly generated with normal distribution. Average and standard deviation of the durations are given by experimental data obtained in cell cultures in exponential growth phase. The target cell division rate and cell cycle parameters are calibrated using *in vitro* measurements. Supplementary Fig. 3 shows that the model fits in exponential growth conditions for both types of target cells.

Cell cycle is split into two phases: G1/S and G2/M phases. Target cells double their mass and volume during the G2/M phase. When mitosis phase ends, the mother cell cycle is reset with new duration parameters to mimic the generation of a first daughter cell; a second daughter cell is created next to the first one with a division plan randomly generated.

When two target cells are entering in physical contact, they adhere while continuing cycling. When a target cell enters in contact with a CTL, they adhere, but target cell stops cycling to enter in defense state. During the defense state, the survival duration of the target cell is reduced at each time step. The survival duration is calibrated using parameter fitting based on experimental data from killing assays.

The reduction of survival duration is directly proportional to the number of CTL the target is in contact with. The total survival duration of a target cell corresponds to the amount of time necessary for one CTL to kill one target cell. During the defense state, CTL within a range of a given distance have a given probability to be disabled at each time step. The target cell can switch back to cycling state if all CTL that are interacting with the target cell get disabled or loose contact. However, when the survival duration is equal to zero, the defense state ends: the target cell dies and disappears from the simulated environment. All parameters corresponding to this state have been established by doing parameter fitting on experimental data from killing assays.

#### Cytotoxic T Lymphocytes

When created, CTLs are starting in a random motion state to randomly explore the environment. If two CTL enter in contact, they do not adhere and interpenetration is solved by the physics engine. Random motion is defined as a Brownian motion with parameters derived from Christophe *et al*.^33^ When a CTL enters in contact with a target cell, they adhere. The CTL switches to an attacking state during which the CTL reduces the survival duration of the target cell. In the meantime, the CTL can be disabled during contact with target cell as described above. This killing activity can be stopped by target cell death or by CTL inactivation. When CTL get disabled, they do not die, but switch back to random motion and remain in this state until the end of the simulation.

#### Model implementation

The model is implemented using C++ 14 programming language. The datasets generated and analyzed in this study, together with generated code, are available upon request.

#### Parameter sampling

The disabling radius *R*_*d*_ and disabling probability *P*_*d*_ parameters of the model were technically complex to obtain experimentally. We have performed a parameter sampling on both these parameters measuring the distance to experimental data at the different E/T ratio available and for both cell lines in the ranges ${R}_{d}\in [50,\,400]$ with steps of 12.5 μm and ${P}_{d}\in [0.00025,\,0.01]$ with steps of 0.00025. The best couple (*R*_*d*_, *P*_*d*_) has been selected using the mean square distance to experimental data on the available E/T ratios (0.01, 0.05, 0.1, 0.5 and 1.0), both cell lines (conventional and melanoma cells) averaged on 10 runs per line and per ratio.

## Supplementary information

Supplementary information

Movie 1

Movie 2
