# Supplementary material for: Sequential adjustment of cytotoxic T lymphocyte densities improves efficacy in controlling tumor growth
Source: Sci Rep. 2019 Aug 23;9:12308. doi: 10.1038/s41598-019-48711-2 (PMC6707257; doi:10.1038/s41598-019-48711-2)
Supplement: Supplementary file 1 — Supplementary information [file 41598_2019_48711_MOESM1_ESM.pdf]

Sequential adjustment of cytotoxic T lymphocyte densities improves efficacy in controlling tumor growth

Roxana Khazen[1,2], Sabina Müller[1], Fanny Lafouresse[1], Salvatore Valitutti[1,3,6*] and Sylvain Cussat-Blanc[4,5,6]

[1]INSERM U1037, Centre de Recherche en Cancérologie de Toulouse (CRCT), Université de Toulouse III-Paul Sabatier, 31057 Toulouse, France. Team: Molecular dynamics of lymphocyte interaction, « Equipe labellisée Ligue Nationale contre le Cancer 2018 »

[2]Present Address : INSERM U1223, Dynamics of Immune Responses Unit, Institut Pasteur, 75015 Paris, France.

[3]Department of Pathology, Institut Universitaire du Cancer-Oncopole de Toulouse, 31059 Toulouse, France.

[4]Institute of Advanced Technologies in Living Sciences, CNRS – UMS3505, Toulouse, France. Team: Onko3D

[5]University of Toulouse, Institute of Research in Informatics of Toulouse, CNRS – UMR5505, Toulouse, France. Team: REVA

[6]These authors are co-senior authors

[*]Corresponding author: salvatore.valitutti@inserm.fr

Supplementary Figure 1

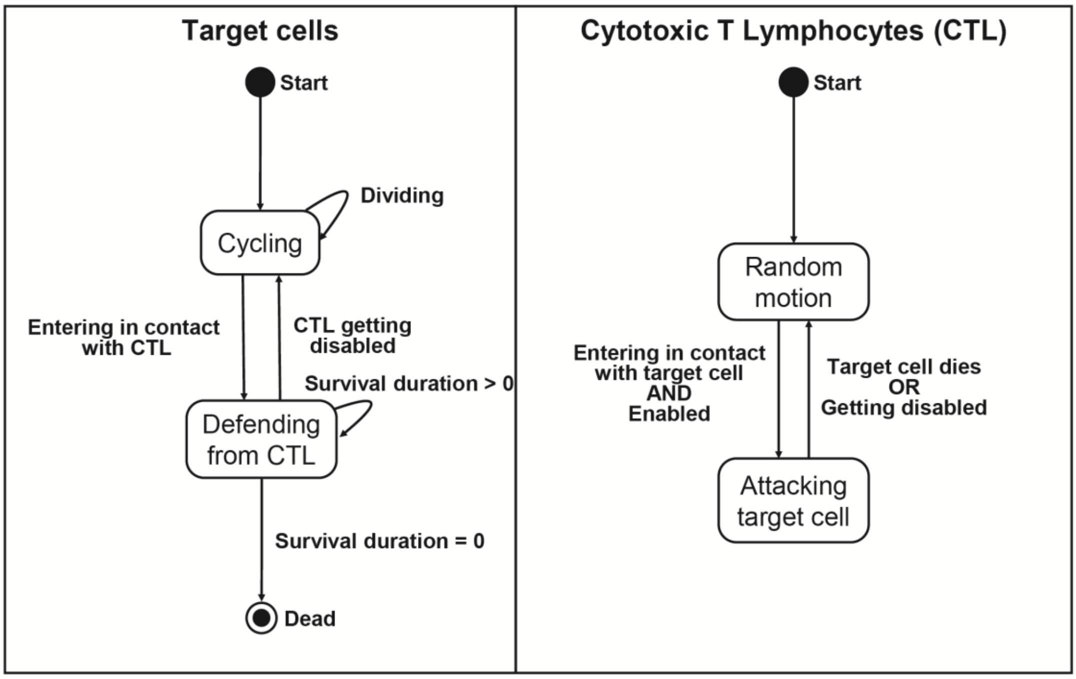

Supplementary Figure 1: State-transition diagram of target cells and CTL behaviors. It describes the life cycle of virtual cells during simulations.

Supplemantary Figure 2

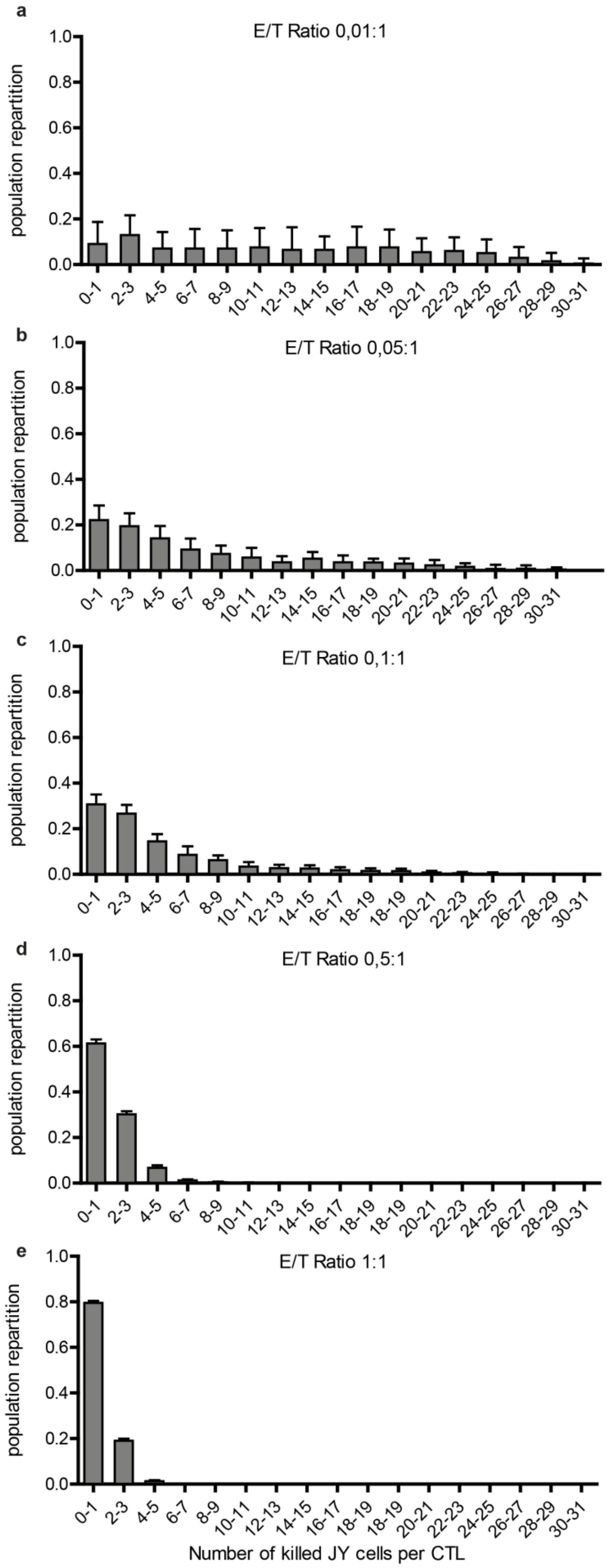

Supplementary Figure 2. Computational modeling highlights deficiency of individual CTL killing capacity at high CTL densities. The panels show the population repartition of CTL killing efficacy at different E/T ratios. The fraction of CTL within the whole population killing a high number of conventional target cells increases with the decrease of the E/T ratio. The E/T ratios simulated in the model are indicated in panels. Results correspond to mean +/- SD of 20 independent runs.

Supplementary Figure 3

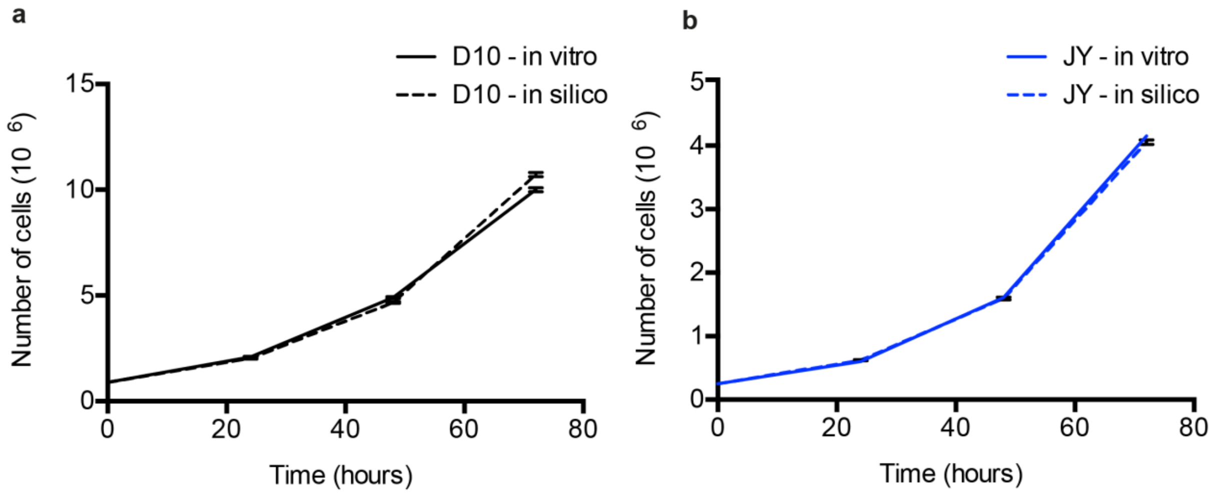

Supplementary Figure 3. Experimental and in silico modeled rate of target cell division. Panel **(A)**, melanoma cells; panel **(B)** conventional target cells.

Supplementary Table1

Parameters used in the model and their values

	Name	Value	Unit	Source
Global	Time step	1.2	Min	Decided
Target cells	Cell cycle duration	MC: 20<br>CC: 17.75	Hours	Experimentally determined in this study
	Cell cycle duration standard deviation	MC: 1<br>CC: 1	Hours	Experimentally determined in this study
	G1S phase duration	MC: 14<br>CC: 13.67	Hours	Experimentally determined in this study
	G2M phase duration	MC: 6<br>CC: 4.08	Hours	Experimentally determined in this study
	Survival duration	MC: 55<br>CC: 11	Min	From Christophe et al 2015
	Disabling radius	87.5	μm	Parameter fitting on experimental data in this study
CTL	Motion speed	8.66	μm/min	From Christophe et al 2015
	Time between direction switch	1	Hour	From Christophe et al 2015
	Disabling probability	0.00275	None	Parameter fitting on experimental data in this study

Supplementary Table2

Paired statistical comparison of CTL efficacy: comparison of sequential CTL addition versus initial addition for various CTL numbers

Protocols	Wilcoxon test time	Wilcoxon p-values
2:1+2:1 vs 4:1	168h (7 days)	2.53e-4
4:1+4:1 vs 8:1	264h (11 days)	2.16e-3
4:1+4:1+4:1 vs 12:1	360h (15 days)	8.81e-5
4:1+4:1+4:1+4:1 vs 16:1	360h (15 days)	1.18e-4

Supplementary movies legends

Movie 1. Example of a simulated interaction at 1:1 E/T ratio

Movie 2. Example of a simulated interaction at 10:1 E/T ratio
